# Case Report: A Case Report and Literature Review of 3p Deletion Syndrome

**DOI:** 10.3389/fped.2021.618059

**Published:** 2021-02-10

**Authors:** Junxian Fu, Ting Wang, Zhuo Fu, Tianxia Li, Xiaomeng Zhang, Jingjing Zhao, Guanglu Yang

**Affiliations:** Department of Pediatric, The Affiliated Hospital of Inner Mongolia Medical University, Hohhot, China

**Keywords:** 3p deletion syndrome, chromosomal disorder, BRPF1 gene deletion, mental developmental retardation, ptosis

## Abstract

**Objective:** The aim of the present study is to explore the clinical and genetic characteristics of 3p deletion syndrome to improve clinicians' understanding of the disease.

**Methods:** The clinical manifestations, process of diagnosis and treatment, and genetic characteristics of an individual case of 3p deletion syndrome were analyzed. CNKI, Wanfang Data, and the Biomedical Literature Database (PubMed) were searched. The search time limit, using “3p deletion syndrome” and “*BRPF1*” as keywords, was from the creation of the database up to June 2020. Related data were reviewed.

**Results:** The proband was a male child with general developmental and intellectual disabilities, special facial features and congenital heart disease. The child was the parents' first pregnancy and first born. Gene microarray analysis showed a 10.095 Mb deletion in the 3p26.3-p25.3 region, resulting in a heterozygous mutation of the *BRPF1* gene; thus, the patient was diagnosed with 3p deletion syndrome. At the time of diagnosis, the child was 1 year of age and was responding to comprehensive rehabilitation training. A total of 29 well-documented cases were found in the literature, of which 19 cases had an onset within 1 year of birth, and mainly manifested with mental and motor development disabilities and abnormal facial features, with different gene deletions, depending on the size and location of the 3p deletion.

**Conclusion:** The genetic test results of the child in this study indicated a heterozygous deletion of the *BRPF1* gene on the short arm of chromosome 3, which was a unique feature of this study, since it was rarely mentioned in other reports of 3p deletion syndrome. The clinical phenotype of this syndrome is complex as it can include intellectual and motor development backwardness, low muscle tone, certain abnormal facial features (low hairline, bilateral ptosis, widely spaced eyes, a forward nose, left ear auricle deformity, a high-arched palate, a small jaw), and the deformation of systems such as the gastrointestinal tract and the urinary tract malformation or symptoms of epilepsy. As clinical manifestations can be relatively mild, the syndrome is easy to miss or misdiagnose. Clinical workers need to be aware of this disease when they find that children have special features, such as stunted growth, low muscle tone or ptosis, and it needs to be diagnosed through genetic testing. Most children are able to develop certain social skills after rehabilitation treatment.

## Introduction

3p deletion syndrome is a rare autosomal and contiguous genomic disorder characterized by the following: intellectual disability; motor developmental delay; unusual facial features (microcephaly, micrognathia, ptosis, long philtrum, low and deformed ears, polydactyly deformity); hypotonia; and other rarer symptoms, including congenital heart disease (CHD), renal and gastrointestinal malformations, autism, congenital hypothyroidism, epilepsy, and tumors (1). The *BRPF1* gene is a chromatin regulator that binds to methylated histone H3 and promotes acetylation. *BRPF1* deletion may lead to intellectual disability and abnormal facial features, such as ptosis (2–9). In the present study, the clinical features, the process of diagnosis and treatment, and the genetic findings of a patient with 3p deletion syndrome were reviewed, and a review of the literature was conducted to summarize the clinical features and genetic characteristics of the syndrome to aid in clinical recognition and identification.

## Clinical Data

The pediatric patient was a 1-year-old male who was the parents' first pregnancy and first born. The patient was born by caesarian section after being unable to enter the pelvic cavity at full term. There was no history of hypoxia or asphyxia. The birth weight was 3.05 kg, and the patient failed to pass the hearing screening at 3, 5, 42 days after birth, and 3 months of age. The parents were non-consanguineous and denied any family history of genetic diseases. There had been no abnormalities in a Down's screening. The mother had a cold during her pregnancy but did not take any medication. There was a history of secondhand smoke exposure but no history of radiation or chemical carcinogen exposure.

**Figure 1:**
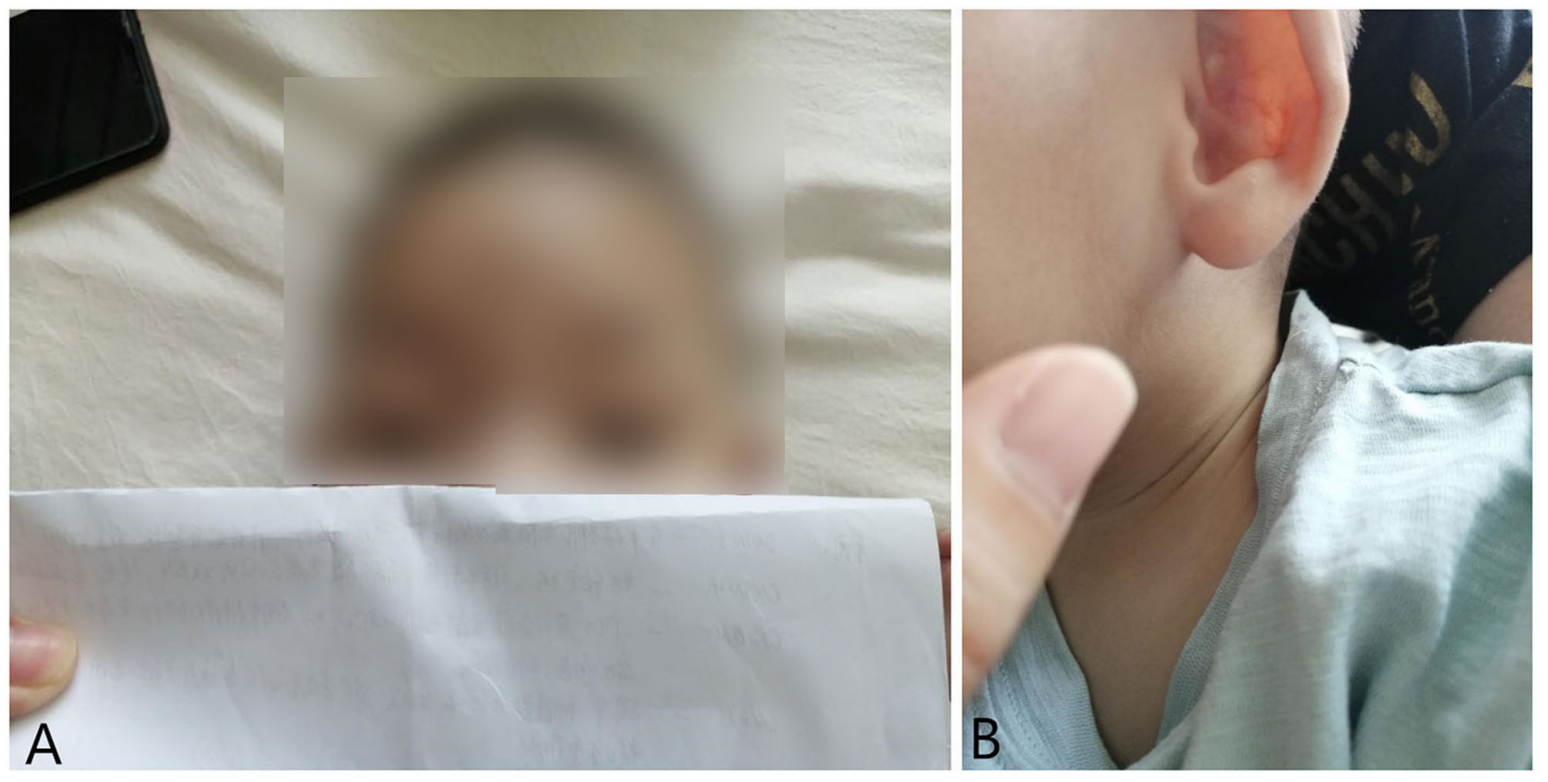
Ptosis **(A)** and auricular deformity **(B)** in the case of 1 year old with 3p deletion syndrome.

Case history: shortly after birth the child started to suffer repeated hiccupping and would vomit more than 10 times a day. The patient was referred to a hospital in Beijing 3 months after birth. A gastrointestinal angiography was performed, which led to the diagnosis of gastroesophageal reflux. Medication was given and the vomiting was relieved at 8 months, but the patient continued to suffer from vomiting when coughing or sneezing. The patient could hold his head erect at 4 months of age, and recognize things and people at 10 months. However, by age 11 months, the patient was no longer able to hold his head erect or sit or climb unassisted. The patient could not walk and could only stand on his toes. The patient could say the words “dad” and “mom” and presented with hyperhidrosis. After being admitted to the hospital, the patient was diagnosed with 3p deletion syndrome after genetic testing. At 1 year of age, the patient was referred to our hospital for rehabilitation training. A physical examination yielded the following observations. The patient was in clear consciousness with average spirit and moderate nutrition. He was 76 cm in height, 8.5 kg in weight, and his head circumference was 44.5 cm. His front hairline was low, and he had bilateral ptosis (drooping eyelids) and wide-set eyes. The pupils were equal in a circle, and the light reflection was sensitive. The patient had a broad nose bridge, forward nostrils, a deformity of the left auricle, a long philtrum, a high-arched palate, and micrognathia (as shown in Figure 1). The teeth were hypoplastic and yellow in color. There was no penetration of the hands. There was no obvious abnormality of the lungs or abdomen and no obvious pathological murmur in the heart valve area. There were no stereotyped movements. The knee reflex and Achilles tendon reflex of the left lower limb were weak, but the tendon reflex of the right lower limb, the bicep reflex of the upper limbs, the triceps reflex, the periosteum reflex of the radius, and the reflex of the abdominal wall were all present, and there was no evident pathological reflex. The patient had hypotonia in the four extremities and uncooperative muscle strength. The patient presented with intellectual disabilities with an inability to recognize strangers or clap hands; further, the patient showed a poor ability to track light and objects.

A number of measurements were made to evaluate the patient's problems. A biochemical analysis showed no obvious abnormalities. The results of the Peabody Developmental Motor Scale were as follows: gesture, three points in the standard score; moving, 1 point in the standard score; physical operation, 0 point in the standard score; grasp, 2 points in the standard score; vision-motion integration, five points in the standard score; for total movement, a development quotient of 51, for coarse movement, a development quotient of 51 and for fine movement, a development quotient of 61. A colored ultrasonography of the heart revealed a congenital heart defect (CHD), namely a ventricular septal defect (peri-membranous) of ~3 mm in size and a left to right shunt at the ventricular level. A video EEG showed an abnormal EEG for a young child with slightly slower background waves during the awake period and spikes and slow waves of the frontal area and midline area (Fz) emitted during sleep. An MRI of the head was normal, but examination of the eyes showed that the lower edge of the pupil was obscured by around 1 mm by both eyelids, giving a diagnosis of bilateral ptosis. With respect to an otolaryngology examination, the patient did not cooperate during the hearing screening, but the child responded to his parents' voices and environmental sounds, indicating that he had no hearing loss. Metabolic screening of the blood and urine revealed no abnormalities.

The patient's clinical manifestations, combined with some well-established auxiliary examinations, indicated a genetic disease, so it was recommended that the family members underwent genetic testing to offer further clarification (Table 1). The genomic DNA of a blood sample was extracted with a Qiagen FlexiGene DNA Kit with approval from the Medical Ethics Committee and family consent. The informed consent forms can be found in the Supplementary Material. An Affymetrix CytoScan750K_Array chromosome microarray chip (Affymetrix, Santa Clara, California, USA) was used to detect the potential copy number variation. DNA extraction and quality control, restrictive enzymatic digestion and labeling, hybridization purification, and scanning were performed, along with other related steps. The genomic variation databases OMIM, HGMD, and ClinVar were used to analyze the array data, and the results were as follows: arr3p26.3-p25.3 (147389-10243084) × 1, 3p26.3-p25.3 was missing 10.095Mb in size. After qPCR verification, heterozygous deletion mutations in exons five and 12 of the *BRPF1* gene were found (as shown in Table 2). The parents did not have the above-mentioned variant, so it was considered a novel variant, and the patient was diagnosed with 3p deletion syndrome.

**Table 1:** Genetic test results.

**Serial number**	**Types of CNV**	**Fragment size**	**Chromosome position**	**Pathogenicity analysis**	**Decipher**	**Region gene**
1	Deletion	10.095Mb	3p26.3-p25.3 (chr3:147,389–10,243,084)*1	Likely pathogenic	–	*ITPR1-AS1, SRGAP3-AS2, SRGAP3-AS4, MIR4790, LOC101927394, IRAK 2, BRPF1, CNTN4-AS1, CRELD1, ARPC4-TTLL3, PRRT3-AS1, GRM7, SSUH2, PRRT3, EMC3, GR M7-AS1, LINC00312, JAGN1, RPUSD3, CP NE9, ARPC4, CNTN6, BHLHE40, THU MPD3, EDEM1, SRGAP3-AS3, LHFPL4, ITPR1, LOC401052, SUM F1, ARL8B, BHLHE40-AS1, CHL1, GRM7-AS2, LINC01266, EGOT, CIDECP, MTM R14, EMC3-AS1, SRGAP3, VHL, LMCD1, LMCD1-AS1, OGG1, TTLL3, IL5RA, CAV3, RAD1 8,IL17RE, BRK1, IL17RC, SETD5, CIDEC, THUMPD3-AS1, CNTN4, TADA3, TRNT1, LRRN1, F ANCD2, CRBN, CHL1-AS2, OXTR, FANCD2OS, CHL1-AS1, CNTN4-AS2, GRM7-AS3, SETMAR, CAMK1*

**Table 2:** Deletion variants of the exons.

**Related gene**	**Location**	**Exon**	**Sequence length**	**Variation type**
*BRPF1*	chr3:9775825-9776423	exon2	599	Gene deletion
*BRPF1*	chr3:9780683-9781642	exon3	960	Gene deletion
*BRPF1*	chr3:9782463-9782625	exon4	163	Gene deletion
*BRPF1*	chr3:9782992-9783123	exon5	132	Gene deletion
*BRPF1*	chr3:9783709-9783855	exon6	147	Delete
*BRPF1*	chr3:9784628-9784937	exon7	310	Gene deletion
*BRPF1*	chr3:9785262-9785585	exon8	324	Gene deletion
*BRPF1*	chr3:9785908-9786192	exon9	285	Gene deletion
*BRPF1*	chr3:9786692-9786839	exon10	148	Gene deletion
*BRPF1*	chr3:9787257-9787614	exon11,12	358	Gene deletion
*BRPF1*	chr3:9787984-9788138	exon13	155	Gene deletion
*BRPF1*	chr3:9788850-9789033	exon14	184	Gene deletion

The treatment the patient was given consisted of a comprehensive rehabilitation program, which included large joint loosening training, Brunstrom training, exercise system training, head control and climbing strength training, four-point kneeling and one knee kneeling, and sitting training. After more than 12 days of rehabilitation, the responses had improved, and the patient could turn over, clap his hands, chase people and objects, grasp objects, sit up for more than half an hour, and stand for around 3 min.

## Discussion

3p deletion syndrome is a rare contiguous genomic disease. To date, no more than 60 cases have been detected globally. The syndrome is mainly caused by deletion of the short arm of chromosome 3.3p. Currently reported cases mainly show 3p25, 3p25-26, and 3p26 deletions. Both terminal and middle deletions in the short arm of chromosome 3 may cause 3p deletion syndrome, though the syndrome is most common in large terminal *de novo* deletions. The retrieved clinical data of 29 cases with 3p deletion syndrome reported in China and in other parts of the world are summarized in Table 3. As shown, 19 cases had an onset within 1 year of age, and four pediatric patients died within 3 months of birth. Of these four cases, three died of unknown causes, and one died from complications of influenza.

**Table 3:** The clinical characteristics and results of the literature review of the 3p deletion syndrome.

**References**	**This patient**	**Wu L et al. (10)**	**Thomas et al**. **(****11****)**		**Dijkhuizen et al**. **(****12****)**		**Helena et al. (13)**	**Helena et al. (13)**	**Pohjolaet al. (14)**	**Pohjola et al. (14)**	**Fernandez et al. (15)**	**Kiraz et al. (16)**	**Shuib et al. (17)**	**Shuib et al. (17)**	**Shuib et al. (17)**	**Shuib et al. (17)**	**Gunnarsson et al. (18)**	**Cristina et al. (19)**	**Peltekova et al. (20)**	**Anupam Kaur et al. (21)**	**Kellogg et al. (22)**	**Omrani et al. (23)**	**Sato et al**. **(****24****)**		**Zhang K et al. (25)**	**Liu X et al. (26)**	**Feng L et al. (27)**	**Zhao X et al**. **(****28****)**			**Philip N et al. (29)**	**Moncla A et al. (30)**	**Colyn B et al. (31)**	**Angelika et al. (32)**
Age of onset	3 month	6 month	6 year-old		6 month		2 month	5 month	5 year-old	10 month	Infancy	Infancy	ND	ND	ND	ND	Infancy	8 year-old	3 month	3 month	6 month	3 month	Infancy		4 month	2d	Infancy	Infancy			Infancy	5 month	16 month	3 years
Motor development retardation	+	+	+		+		+	+	+	+	+	+	–	+	+	+	+	–	+	+	+	+	+		+	+	+	+			+	+	+	+
Intellectual disability	+	+	+		+		+	+	+	+	+	+	–	+	+	+	+	+	+	+	+	ND	+		+	+	+	+			+	+	+	+
Hypotonia	+	+	+		ND		+	+	+	–	–	+	–	ND	ND	ND	+	–	+	ND	+	ND	+	+		–	+	+	+			+	+	+
Ptosis	+	+	+		–		–	–	–	–	–	+	–	+	+	+	+	–	+	–	–	+	+		+	–	–	–			+	+	+	–
Long philtrum	+	+	–		–		+	+	+	–	+	–	–	–	–	–	+	–	–	–	+	+	+		–	–	–	+			+	+	+	+
Wide nose	+	ND	+		–		+	+	–	–	–	+	–	–	–	–	+	–	–	+	+	+	+		+	+	–	+			+	+	+	+
Micrognathia	+	ND	–		–		–	+	–	–	+	–	–	+	+	+	–	–	–	+	–	+	+		+	+	–	+			+	+	+	–
hearing abnormality	–	ND	–		–		–	+	–	–	+	ND	–	–	–	–	–	–	–	ND	ND	ND	–		+	ND	+	ND			ND	ND	–	–
congenital heart disease	+	+	–		ND		–	–	–	ND	–	+	–	+	–	–	+	–	+	ND	–	ND	–		+	+	+	+			–	ND	–	–
Gastrointestinal abnormalities	–	–	–		ND		+	ND	–	ND	ND	–	–	–	–	–	–	–	+	ND	–	ND	+		–	–	–	+			ND	ND	ND	–
Gene deletion	3p26.3–p25.3 about 10.095Mb deleted (Containing BRPF1)	3p25.3 about 10Mb deleted	3p26.2–3p26.3	deleted (Containing CNTN4)	3p26.2–3pter	About 4Mb deleted (Containing CHL1, CNTN4 and CRBN)	3p25.2 Large segment deleted (Containing SLC6A11)	3p25.3p26.3 about 10Mb deleted (Containing CHL1, ATP2B2)	3p25.3 about 8.99Mb deleted	3p26 about 1.1Mb deleted (Containing CHL1)	3p26.2 about 4.5Mb deleted (Containing CNTN4)	3p25.3–pter about 8.727 Mb deleted	3p26.1–pter about 8.60 deleted	3p25.3–pter about 11.50Mb deleted (Containing FANCD2, VHL, IRAK2, GHRL)	3p25.3–p26.1 about 6.30Mb deleted (Containing FANCD2, VHL, IRAK2, GHRL)	3p25.3–pter about 10.90Mb deleted (Containing FANCD2, VHL, IRAK2, GHRL)	3p25.3–p26.1 about 1.6Mb deleted (Containing CAV3, OXTR and SRGAP3/MEGAP)	3p26.3 about 555.4 Kb deleted (Containing CHL1)	3p25.3 about 643kb deleted (Containing THUMP3, SETD5, LHFPL4, MTMR14, CPNE9, BRPF1, OGG1, CAMK1, TADA3, ARPC4, TTLL3 and RPUSD3)	3p25–p26.34 deleted (Maybe containing CHL)	3p25.3 about 684kb deleted (Containing SETD5, THUMPD3 and LOC440944)	3p23–p26 deleted	3p26.3–25.3 about 11.0Mb deleted	(Containing VHL)	3pterp25.3 about 9.918 Mb deleted	3p26.1pter about 4.190 Mb deleted	3p26.3–pter about 2.16Mb Microdeletions (containing 7 pathogenic genes)	3p25 about	3327 kb deleted (Containing SETD5,	*VHL* and FANCD2)	3p25–pter deleted	3p25–3pter deleted	3p25–p26 about 4.5Mb deleted	3p 26.1–p 25.3about 1.24Mb deleted (Containing *LOC100288428*,*LMCD1, LINC00312, C3orf32, CAV3, OXTR, RAD18, SRGAP3,THUMPD3, LOC440944, SETD5*)
Prognosis	Survival	Survival	Survival		Survival		Survival	Survival	Survival	Survival	Survival	Survival	Survival	Survival	Survival	Survival	Survival	Survival	Dead	Dead	Survival	Survival	Survival		Survival	Dead	Survival	Survival			Dead	Survival	Survival	Survival

*+, yes; –, no; ND, undescribed*.

3p deletion syndrome was first reported by Verjaal and De Nef in 1978 (10). After more than 40 years of studies, the main clinical phenotypes of the disease have been identified as follows: delayed growth and development, intellectual disability, hypotonia, micrognathia, ptosis, wide nose bridge, long philtrum, low ear position, deformed ears, polydactyly deformity, hearing abnormalities, CHD, renal abnormalities, syndactylism, gastrointestinal abnormalities, and scoliosis (12, 13). The incidence of the main symptoms is 95% for growth retardation, 79% for intellectual disability, 61% for hypotonia, and 42% for ptosis. The incidence of other syndromes is 43% for CHD and 29% for hearing abnormalities. In the present case, the pediatric patient had the main manifestations of delayed growth and development, intellectual disability, hypotonia, micrognathia, ptosis, a wide nose bridge and auricle deformity, and CHD. As the patient did not cooperate with the hearing test, follow-up observation was required to check their hearing.

Exon Slim + CMA chromosome chip testing revealed a 10.095 Mb deletion in 3p26.3-p25.3 and a heterozygous deletion in exons five and 12 in the *BRPF1* gene. The patient had most of the main clinical phenotypes, as well as other features, such as CHD. *BRPF1* is a chromatin regulator with a PHD domain, bromouracil domain, PWWP domain, and two enhancers (14). PHD is responsible for the activation of three histone lysine acetyltransferases (MOZ, MORF, and HBO1) while bromouracil is used for acetyl-lysine recognition, and the PWWP domain is used for specific interactions with methylated histone H3. *BRPF1* forms a complex with MOZ, MORF, and HBO1, and acts as a scaffold to bridge subunit interactions, stimulate acetyltransferase activity, and limit substrate specificity (15). Lysine acetyltransferase 6A (KAT6A) and its secondary chain, KAT6B, form chemical complexes with *BRPF1* for the acetylation and propionylation of histone H3 at lysine 23 (H3K23). When missing *BRPF1*, variant histone H3 acetylation in H3K23 and propionyl defects affect chromatin modification after translation. The lack of *BRPF1* can also lead to multiple gene (such as Robo3 and Otx1) transcription reduction (16, 17), the loss of neuronal migration and neural tube closure, the control of transcriptional regulation, and it can cause neurodevelopmental disorders, which can result in intellectual disability. In this case, the child had intellectual disability, and the genetic test results suggested the deletion of *BRPF1* gene. Combined with the previous research results, it was proved that the intellectual retardation of this child was caused by the deletion of the BRPF1 gene. In addition, the *BRPF1* gene plays a role in craniofacial development, and it has also been confirmed that a lack of *BRPF1* can cause ptosis and (or) narrow palpebral fissure (18, 19). According to our statistics, the ptosis occurrence rate is about 40%. In hospitalized cases, during the perfect eye exam, a bilateral palpebral edge of shaded pupil of about 1 mm is considered to be a sign of ptosis. We consider such cases to be *BRPF1* ptosis, resulting from gene deletion (20–24). In the present case, the heterozygous deletion of *BRPF1* appeared to have led to intellectual disability and ptosis, but not to hearing abnormalities or renal and gastrointestinal malformations. The other clinical phenotypes, such as hypermobility, are thought to have been caused by the absence of large segments of the short arm of chromosome 3.

Previous studies have shown that the size of a deletion can vary, ranging from one to several trillion bases. Furthermore, most deletions are *de novo* and occur in a family for the first time, though familial cases have also been shown to occur. Not all patients with deficits have the typical features of the syndrome, and some have only mild symptoms or are even completely free of them (25–28, 33). Most patients with 3p deletion syndrome have a moderate or higher intellectual disability. Initially, it was thought that the *CHL1* located at 3p26.3 (at the 213649-426090 bp of the p telomere) was associated with neurodevelopment (34), though later genetic studies in familial patients found that a 1.1 Mb deletion covering the *CHL1* alone was insufficient to produce a clinical phenotype of the 3p deletion syndrome (35). Genetic analysis of a complex chromosome 3 aberration leading to 3p deletion syndrome conducted by Dijkhuizen and Essen suggested that the main features of 3p deletion syndrome are caused by *CNTN4* and *CRBN* deletions and that *CHL1* could be a candidate gene for non-specific developmental delays in the disease (36). As more cases have been discovered, more genes have been found to be linked to intellectual disability. Salwati et al. narrowed down the candidate critical region for intellectual disability to a region ~950 kb in size containing *SRGAP3*. They therefore concluded that this gene may be the key gene for intellectual disability (37). Peter et al. further reduced the region containing the gene to 485.78 kb (35). However, an *SRGAP3* deficiency cannot explain all cases of intellectual disability in 3p deletion syndrome. Gregory reported on a patient with intellectual disability and dysmorphic features and co-analyzed the patient's genes with those of three other reported cases. Here, it was found that the patients shared a missing region, ~124 kb in size, that contained the genes of *THUMPD3, SETD5*, and *LOC440944* (22), which suggested that these genes may correlate with intellectual disability. Xiaofeng et al. reported a case of 3p25.3p25.2 chromosomal heterozygous deletion with gastrointestinal malformation and intellectual disability in a case with 3p deletion syndrome with a missing gene size of 3327 kb containing *SETD5, SRGPA3*, and *SEC13*. The deletion of *SETD5* and *SRGPA3* was associated with intellectual disability, while the deletion of *SEC13* may have been responsible for gastrointestinal developmental abnormalities (28). Although recent studies have suggested that *SETD5* and *SRGPA3* are the key genes involved in intellectual disability in 3p deletion syndrome, the genetic test results in the present case showed that *BRPF1* deletion may also cause intellectual disability.

The patient in the present study also had CHD. The candidate genes proposed for CHD in the present study are *SEC13R, SLC6A11*, and *CAV3*. Genetic analysis of a further patient with CHD by Helena showed a deletion of *SLC6A11*, which suggests that it may a candidate gene (13). Cecilia and Cathrine showed that *CAV3* haploinsufficiency may cause CHD in 3p deletion syndrome (18). Meanwhile, Timothy et al. proposed locating the cardiac development gene at the junction of D3S1585 and D3S1317, in the region that includes *SEC13R*, and suggested that it may be a candidate gene for CHD (36–39). However, Elaine and Matthew reported localizing the CHD gene to the D3S1263 to D3S3594 region, in which candidate genes for CHD proposed in previous studies, such as *CAV3* and *SEC13R*, are excluded (37). Salwati analyzed 3p deletion syndrome by microarray and mapped the candidate intervals for CHD susceptibility sites to ~200 kb intervals. On the other hand, some known candidate genes, such as *CAV3* and *SEC13R*, were shown to be outside the target interval. This could be due to the fact that genes beyond the critical interval for CHD may have altered expression due to changes in the deletion of the regulatory regions (29). The CHD-related gene deletions suggested in previous studies were not identified in the present case, which suggested that the development of CHD in the present case may correlate with an absence of large segments of the short arm of chromosome 3.

In addition, some pediatric patients with 3p deletion syndrome may manifest hearing impairment and autistic tendencies, which may correlate with *ATP2B2* (38) and *CNTN4* deficiency (11, 39), respectively. A small number of patients develop tumors. Sato reported the first case of 3p deletion syndrome combined with cerebellar angioblastoma in a child, which was mainly caused by a *VHL* gene deletion (30). Some patients with 3p deletion syndrome may also have other chromosomal translocations that lead to a more severe clinical phenotype (31, 32), though genetic microarray analysis can only detect deletions and duplications. The diagnosis of other forms of mutations, including point mutations, inversions, or translocations, requires other tests, such as chromosomal karyotyping.

No abnormalities of the kidney and gastrointestinal tract were found in the present case, but the electroencephalogram showed abnormal discharges. The child did not cooperate with the hearing screening, so auditory disability could not be confirmed. Future follow-up observations are needed to watch for rare manifestations, such as epilepsy.

## Conclusion

At present there is still little Chinese research into 3p deletion syndrome, but it is known that the exact location of the deletion can vary and different genes can have different clinical phenotypes. This study describes a case of a typical phenotype of 3 p deletion syndrome, exhibiting intellectual and motor development backwardness, low muscle tone, CHD and ptosis. Genetic test results found a heterozygous mutation of the *BRPF1* gene on the short arm of chromosome 3. After more than 2 months of rehabilitation training, the child showed significant growth and progress with their physical responses. Follow-up treatment continued to adhere to the rehabilitation training and was aimed at developing the child's social skills. This case report is expected to provide more references for clinicians to identify the disease. Clinicians should consider 3p deletion syndrome when they are exploring a diagnosis for children with stunting, intellectual disability, ptosis and other special facial manifestations, and the syndrome can be confirmed by means of chromosomal karyotype analysis and gene diagnosis. It has been shown that early rehabilitation therapy and other therapeutic interventions can improve the quality of life of children with this chromosome abnormality.

## Data Availability Statement

The original contributions generated for the study are included in the article/supplementary material, further inquiries can be directed to the corresponding author.

## Ethics Statement

The studies involving human participants were reviewed and approved by Ethics Committee of The Affiliated Hospital of Inner Mongolia Medical University. Written informed consent to participate in this study was provided by the participants' legal guardian/next of kin. Written informed consent was obtained from the individual(s), and minor(s)' legal guardian/next of kin, for the publication of any potentially identifiable images or data included in this article.

## Author Contributions

JF and GY conceived the idea, conceptualized the study, and drafted the manuscript. TW collected the data. ZF and TL analyzed the data. XZ and JS reviewed the manuscript. All authors read and approved the final draft.

## Conflict of Interest

The authors declare that the research was conducted in the absence of any commercial or financial relationships that could be construed as a potential conflict of interest.
