# Supplementary figures and images for: Case Report: A Case Report and Literature Review of 3p Deletion Syndrome
Source: Front Pediatr. 2021 Feb 10;9:618059. doi: 10.3389/fped.2021.618059 (PMC7902511; doi:10.3389/fped.2021.618059)

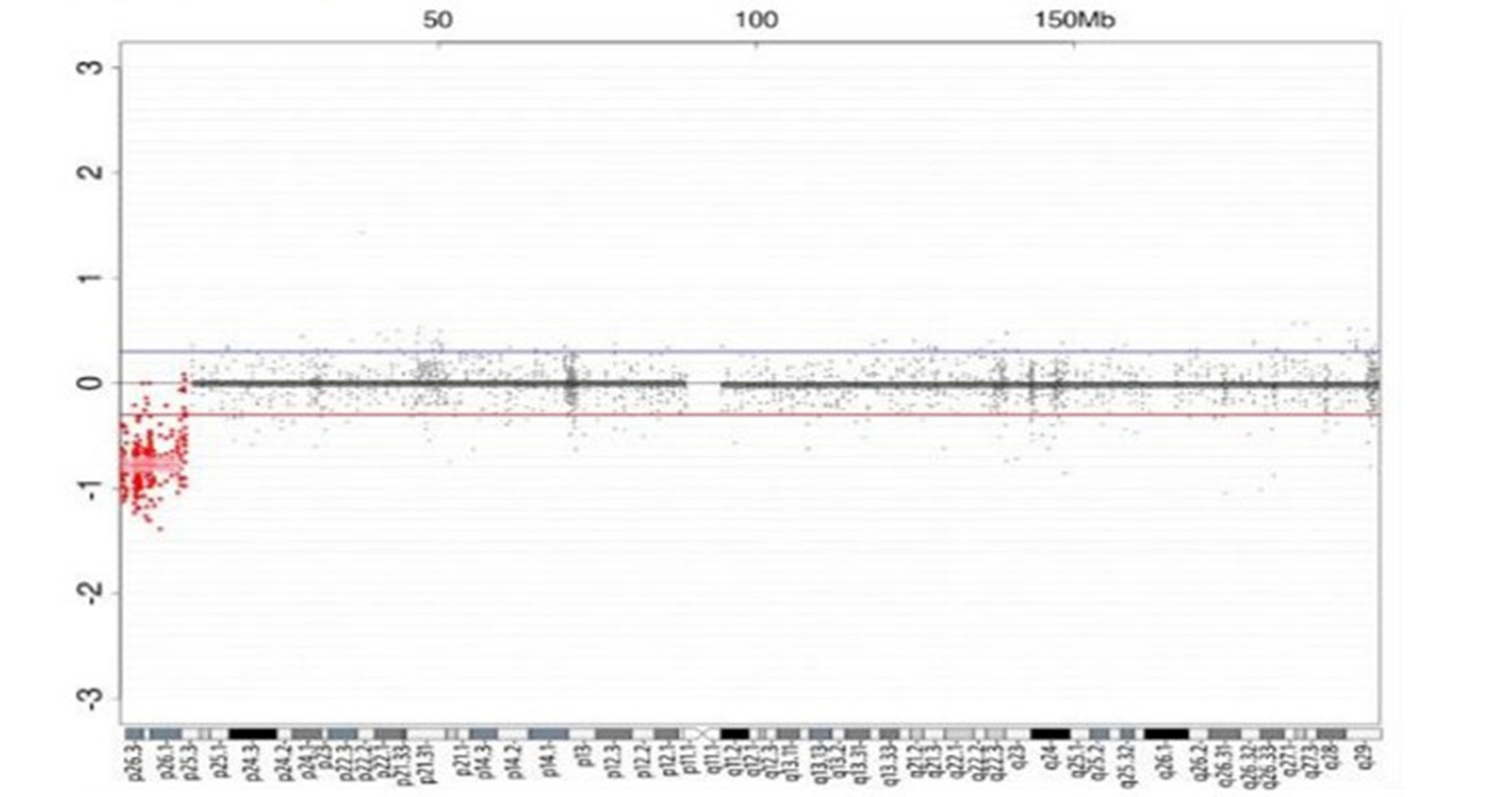

Supplement: Supplementary file 1 [file Image_1.JPEG]

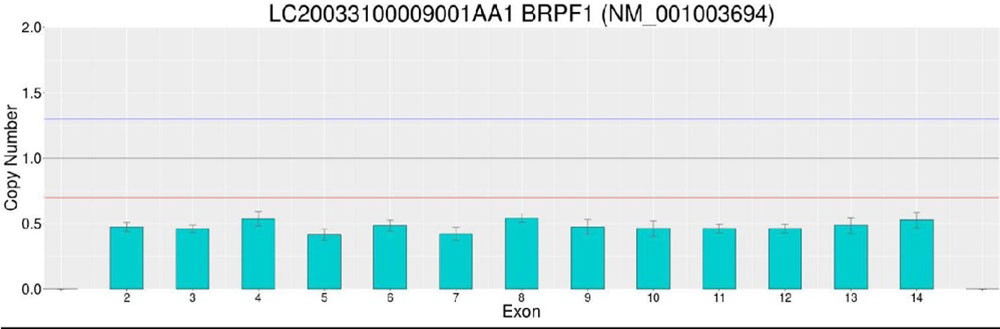

Supplement: Supplementary file 2 [file Image_2.JPEG]

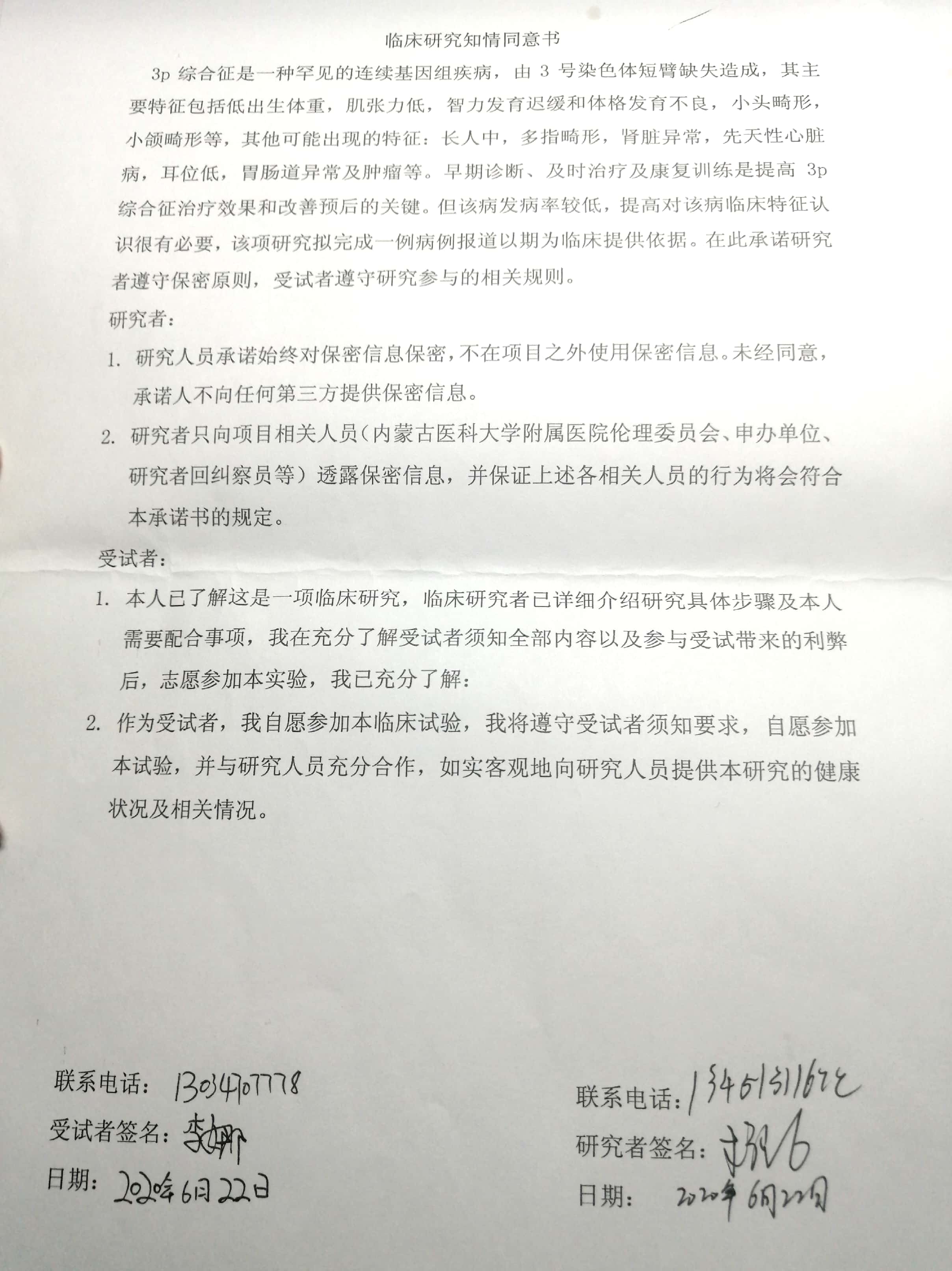

Supplement: Supplementary file 3 [file Image_3.JPEG]
