# Supplementary material for: Case Report: A Case Report and Literature Review of 3p Deletion Syndrome
Source: Front Pediatr. 2021 Feb 10;9:618059. doi: 10.3389/fped.2021.618059 (PMC7902511; doi:10.3389/fped.2021.618059)
Supplement: Supplementary file 4 [file Table_1.DOCX]

Rehabilitation schedule

| Rehabilitation location | Affiliated Hospital of Inner Mongolia Medical University | Beijing Boai Hospital |
| --- | --- | --- |
| Rehabilitation days | 21 days | 51 days |
| Content of rehabilitation | Large joint looseness training, Brunstrom training, motion system training, head control and crawl training, four point kneeling and one knee kneeling, sitting training | Upper limb training (playing with toys to train grasping and coordination), lower limb training (training station, climbing), language training |
| Effects of rehabilitation | Improved reaction, able to turn over, able to clap hands, able to chase people and things, able to grasp things, able to sit for more than half an hour, able to stand for about 3 minutes | My reaction was better. I was able to chase people and things. I had the sense to walk, and when I saw my grandparents, I would ask for hugs |
